# Toward a Disease-Modifying Therapy of Alpha-Synucleinopathies: New Molecules and New Approaches Came into the Limelight

**DOI:** 10.3390/molecules26237351

**Published:** 2021-12-03

**Authors:** Mae Upcott, Kirill D. Chaprov, Vladimir L. Buchman

**Affiliations:** 1 School of Biosciences, Cardiff University, Museum Avenue, Cardiff CF10 3AX, UK; UpcottMR@cardiff.ac.uk; 2 Institute of Physiologically Active Compounds Russian Academy of Sciences (IPAC RAS), 1 Severniy Proezd, Chernogolovk, 142432 Moscow, Russia; chapkir@gmail.com; 3 Belgorod State National Research University, 85 Pobedy Street, 308015 Belgorod, Russia

**Keywords:** alpha-synuclein, therapeutic/therapy, biomarker, rodent models, Parkinson’s disease, translational research

## Abstract

The accumulation of the various products of alpha-synuclein aggregation has been associated with the etiology and pathogenesis of several neurodegenerative conditions, including both familial and sporadic forms of Parkinson’s disease (PD) and dementia with Lewy bodies (DLB). It is now well established that the aggregation and spread of alpha-synuclein aggregation pathology activate numerous pathogenic mechanisms that contribute to neurodegeneration and, ultimately, to disease progression. Therefore, the development of a safe and effective disease-modifying therapy that limits or prevents the accumulation of the toxic intermediate products of alpha-synuclein aggregation and the spread of alpha-synuclein aggregation pathology could provide significant positive clinical outcomes in PD/DLB cohorts. It has been suggested that this goal can be achieved by reducing the intracellular and/or extracellular levels of monomeric and already aggregated alpha-synuclein. The principal aim of this review is to critically evaluate the potential of therapeutic strategies that target the post-transcriptional steps of alpha-synuclein production and immunotherapy-based approaches to alpha-synuclein degradation in PD/DLB patients. Strategies aimed at the downregulation of alpha-synuclein production are at an early preclinical stage of drug development and, although they have shown promise in animal models of alpha-synuclein aggregation, many limitations need to be resolved before in-human clinical trials can be seriously considered. In contrast, many strategies aimed at the degradation of alpha-synuclein using immunotherapeutic approaches are at a more advanced stage of development, with some in-human Phase II clinical trials currently in progress. Translational barriers for both strategies include the limitations of alpha-synuclein aggregation models, poor understanding of the therapeutic window for the alpha-synuclein knockdown, and variability in alpha-synuclein pathology across patient cohorts. Overcoming such barriers should be the main focus of further studies. However, it is already clear that these strategies do have the potential to achieve a disease-modifying effect in PD and DLB.

## 1. Introduction

The pathological aggregation of alpha-synuclein has been implicated in the etiology, pathogenesis and progression of several neurodegenerative diseases, including familial and sporadic forms of Parkinson’s disease (PD), dementia with Lewy bodies (DLB), and multiple system atrophy (MSA), that have coalesced into a group of alpha-synucleinopathies [1,2]. Therefore, the inhibition of alpha-synuclein aggregation and the spreading of aggregation pathology across the nervous system is considered a suitable target for novel disease-modifying therapeutics for these diseases.

### 1.1. Pathological Aggregation of Alpha-Synuclein

Alpha-synuclein is highly abundant in most types of neurons and is predominantly localized in their presynaptic terminals, where it acts as a modulator of various molecular processes involved in synaptic neurotransmission. As a natively unfolded or intrinsically disordered protein (IDP), alpha-synuclein has a high propensity to aggregate. A multistep process of alpha-synuclein aggregation includes nucleation, elongation/fibril growth, secondary nucleation, and, finally, the assembly of mature filaments that, in vivo, form various types of pathological inclusions; these steps were intensively studied in vitro, in cultured cells and in the native nervous system of experimental animals; the results of these studies are summarized and discussed in multiple reviews (for example, see [3,4,5,6,7]). The intermediates of alpha-synuclein aggregation, e.g., oligomers and protofibrils, are believed to be the most important species from the pathology perspective as they exert neurotoxicity and are efficient seeds of further aggregation.

### 1.2. The Neuroanatomical and Cell-to-Cell Propagation of Alpha-Synuclein Aggregation Pathology

**Figure 1:**
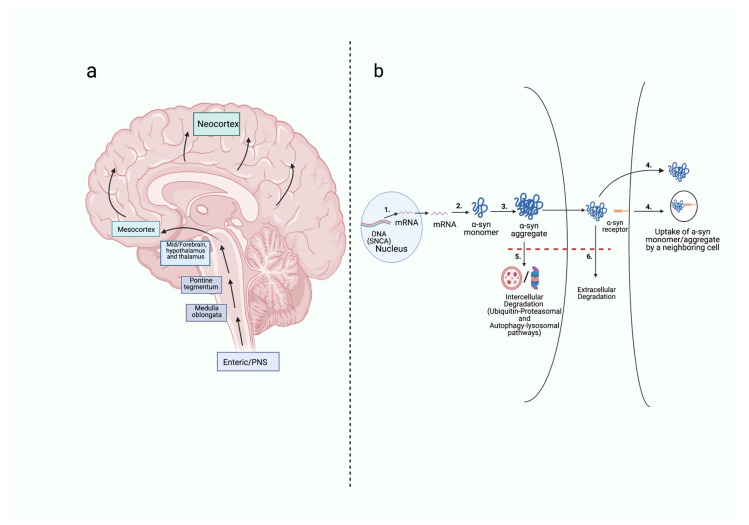
The neuroanatomy of alpha-synuclein aggregation pathology propagation from the enteric nervous system, and the cellular mechanism of this propagation. (**a**) The neuroanatomical propagation of alpha-synuclein throughout the brain is currently best described by the Braak hypothesis. The spread of alpha-synuclein occurs in a caudo-rostral manner, with initial pathological aggregation occurring in the enteric nervous system. Braak stage 1 is characterized by alpha-synuclein accumulation in early brain areas, including the dorsal motor nucleus of the vagus nerve (medulla oblongata) and the olfactory bulb. Braak stage 2 is associated with the involvement of the raphe nuclei and medullary reticular formations (medulla oblongata) and the pontine tegmentum. Braak stage 3 involves alpha-synuclein aggregation in the substantia nigra pars compacta (SNpc) and basal forebrain areas. Braak stage 4 is defined by the neurodegeneration of the SNpc and aggregation observed in the mesocortex (e.g., amygdala). At Braak stage 5, alpha-synuclein pathology can begin to be identified in neocortical areas (e.g., frontal, parietal, and temporal lobes). Finally, Braak stage 6 relates to the most advanced stage of disease progression, with widespread neurodegeneration and widespread neocortical alpha-synuclein accumulation [8]. (**b**) The cellular propagation of alpha-synuclein occurs in a prion-like manner of transmission. The spread of pathological alpha-synuclein in the enteric, peripheral, and central nervous systems is dependent upon six key components, depicted in the above figure: gene transcription (1), alpha-synuclein translation (2), alpha-synuclein aggregation (3), and alpha-synuclein uptake into the neighboring neurons (4), as well as insufficient intracellular (5) and extracellular (6) degradation. This figure was created with BioRender.com.

A widely accepted model of PD progression states that the pathological alpha-synuclein aggregation cascade most often starts in the neurons of the gastrointestinal system (enteric nervous system) or olfactory bulb (central nervous system), then spreads throughout neural tissue in a cell-to-cell prion-like manner (illustrated in Figure 1). In a landmark paper, Braak et al. [8] devised a model that described the propagation of alpha-synuclein pathology in PD, based upon six stages. The Braak hypothesis received support from experiments that used rodent models, as well as from clinical data. For example, vagotomy in both rodent models [9] and clinical cohorts [10] mitigates alpha-synuclein propagation and reduces PD risk. However, the Braak hypothesis is an imperfect model for alpha-synuclein propagation, with cases not showing a caudo-rostral direction of propagation, and correlation with symptomatology being highly variable [11]. However, the Braak stages remain the most widely accepted model to describe the propagation of alpha-synuclein at a neuroanatomical level in PD.

The Braak hypothesis is dependent upon the notion that alpha-synuclein propagation occurs in a prion-like manner. This mechanism of cell-to-cell transmission has been validated in several rodent alpha-synuclein aggregation models [12,13,14,15,16,17,18,19,20]. Therefore, this literature review will assume that the cell-to-cell transmission of pathological alpha-synuclein in PD is dependent upon *SNCA* gene transcription, alpha-synuclein production, alpha-synuclein aggregation and alpha-synuclein uptake into neighboring neurons, in addition to insufficient alpha-synuclein intracellular and extracellular degradation processes.

### 1.3. Pathological Consequences of the Accumulation of Products of Alpha-Synuclein Aggregation

The pathogenicity of this protein aggregation can be attributed to the ability of alpha-synuclein to interfere with multiple and broad cellular mechanisms. These include synaptic dysfunction, dopaminergic cell death in the substantia nigra pars compacta (SNpc), mitochondrial dysfunction, endoplasmic reticulum (ER) stress, neuroinflammation, and the impairment of pathological protein aggregate clearance/degradation via the proteasomal/autophagic pathways. Alpha-synuclein has been shown to be involved in synaptic vesicle fusion/trafficking processes, particularly in the facilitation of presynaptic neurotransmitter release by binding to the synaptobrevin-2 protein, a vesicular component of the SNARE (soluble N-ethylmaleimide-sensitive factor attachment protein receptor) complex [21]. It has been hypothesized that pathological alpha-synuclein accumulation leads to sequestration in aggregates, with the consequent depletion of the pool and the loss of function associated with normal physiological alpha-synuclein, contributing to alterations in neurotransmission that are associated with the progression of synucleinopathies [22,23,24,25]. Oligomeric alpha-synuclein aggregates contribute to the multiple pathological processes that are associated with mitochondrial dysfunction [26,27,28]. This is particularly relevant considering the fact that dopaminergic neurons are highly susceptible to the consequences of mitochondrial dysfunction [29]. It has also been shown that ER stress, induced by A53T alpha-synuclein, can further contribute to increased reactive oxidative species (ROS) production. This can be primarily attributed to an engagement of the unfolded protein response [30] and to increases in cytosolic calcium [31]. Additionally, it has been suggested that increased ROS production can further contribute to alpha-synuclein accumulation [32]. Two main intracellular systems are involved in the degradation of various alpha-synuclein species: the ubiquitin-proteasome system (UPS), degrading physiological alpha-synuclein monomers and small oligomers [33], and the autophagosome-lysosome pathway (ALP), involved in the clearance of larger, mainly pathological alpha-synuclein aggregates [34]. It is now well established that alpha-synuclein accumulation attenuates these mechanisms and, thus, compromises alpha-synuclein clearance/degradation. Alpha-synuclein aggregation also recruits microglia involvement, which causes neuroinflammatory processes that can further potentiate alpha-synuclein accumulation [35].

Considering the fact that prion-like propagation and the subsequent intercellular pathological consequences of alpha-synuclein aggregation are major pathogenic contributors to the development and progression of PD, it is evident that alpha-synuclein is an appropriate therapeutic target for a novel disease-modifying treatment for PD. The intracellular concentration of both monomeric alpha-synuclein and various products of its aggregation is an important factor affecting the pace of propagation of aggregation pathology and the degree of its spread in the nervous system, as well as the manifestation of neurotoxicity and, as a result, the progression of the disease. Therefore, the development of treatments capable of reducing these concentrations in neurons and/or glial cells is as important as those that target the actual process of alpha-synuclein aggregation or the cell-to-cell transmission of aggregation seeds.

## 2. Post-Transcriptional Targeting of Alpha-Synuclein Production

### 2.1. Strategies to Achieve Post-Transcriptional Alpha-Synuclein Knockdown

One widely accepted hypothesis is that therapeutically decreasing the production of alpha-synuclein monomers will reduce the ability of such monomers to undergo the pathological oligomerization and aggregation that are observed in PD. One proposed strategy to decrease alpha-synuclein production is to reduce the level of the encoded mRNA using RNA interference (RNAi) technologies, with both shRNA (short hairpin RNA) and siRNA (small interfering RNA) being able to degrade targeted mRNA sequences [36]. Sapru et al. [37] provided early evidence of the potential utility of such a strategy by demonstrating that a lentivirus-based shRNA that targets human alpha-synuclein-encoding mRNA could indeed successfully decrease alpha-synuclein production in a human dopaminergic cell line, SH-SY5Y, and in rats expressing human protein. Similarly, the AAV (adeno-associated virus)-mediated delivery of shRNA targeting rat alpha-synuclein-encoding mRNA led to a 35% reduction in alpha-synuclein production in the rat brain [38]. Furthermore, it was shown that such an administration of shRNA alleviated motor deficits and also reduced striatal dopaminergic loss in the rotenone rodent model. Importantly, no motor impairments or changes in nigrostriatal morphology and tyrosine hydroxylase (TH) expression were observed in wild-type mice administered with the shRNA. However, the level of dopamine in the striatum of these animals was slightly reduced [38]. The potential efficacy of siRNA in non-human primates has also been shown [39]. In this study, a 21-base pair siRNA duplex against squirrel monkey alpha-synuclein-encoding mRNA was administered unilaterally to the left SNpc in three monkeys. It was shown that this siRNA molecule led to the successful lowering of alpha-synuclein expression by 50% in dopaminergic neurons of the targeted brain area, compared to the untreated side. Furthermore, no adverse effects or toxicity were reported. Unfortunately, the sponsors later stopped funding the research, and the further development of this therapeutic, including investigating its suitability for Phase I clinical trials, ceased. Interestingly, other siRNA-based molecules have also shown promise. For instance, it was shown that the administration of naked siRNA to the mouse hippocampus led to improved resistance to endo/exonuclease degradation and, thus, could achieve a local 70% reduction of *SNCA* expression for up to three weeks after administration [40].

Antisense oligonucleotide (ASO) strategies have also been investigated [41,42]. Alarcón-Arís et al. [43] employed the intranasal delivery of an ASO targeting *SNCA* in conjunction with a triple monoamine transporter blocker (indatraline—IND-1233-ASO). This compound aimed to reduce alpha-synuclein expression in the monoaminergic neurons of brainstem areas (e.g., SNpc), which typically show early pathological alpha-synuclein aggregation, according to the Braak hypothesis [8]. The team observed the decreased expression of endogenous alpha-synuclein mRNA in the brainstem areas, with increases in dopaminergic and serotonergic signaling without neurodegeneration. The authors claim that ASO technologies have a reduced molecular weight, do not require a hybridization stage in production (ASO is a single-stranded oligonucleotide) and reduced toxicity due to bypassing interactions with the RNA-induced silencing complex (RISC), compared to siRNA technologies [43]. This assumption may indeed be correct, with Phase I–IIa clinical trials of an intrathecally administered ASO therapy against the mutant huntingtin sequence reporting a tolerable safety outcome and dose-dependent decreases in mutant huntingtin [44]. Promisingly, it has recently been shown that the successive administration of IND-1233-ASO over 28 days (admittedly, via an intracerebroventricular route) reduced alpha-synuclein production in the SNpc and improved DA signaling in an A30P+A53T alpha-synuclein transgenic aggregation model. However, no changes in performance on motor and behavior batteries were observed [45]. This study proves that IND-1233-ASO has the ability to decrease human alpha-synuclein levels in specifically targeted, PD-relevant brain areas. The fact that IND-1233-ASO did not improve phenotypic presentation is troublesome; however, the authors suggest that this may be due to incorrect dosage or pathology in off-target areas [45]. Interestingly, an amino-bridged nucleic acid (AmNA)-modified ASO showed greater intercellular stability, while retaining the ability to lower alpha-synuclein mRNA and protein levels in human cultured cells, as well as in the human alpha-synuclein aggregation rodent model, TH-SNCA-140. Furthermore, intracerebrally administered AmNA-modified ASO led to improvement in animal motor behavior (e.g., wire suspension test) in the Thy-1-alpha-synuclein rodent model, indicating a therapeutic reduction in motor deficits [46]. It could be argued that this ability to improve phenotypic presentation in the rodent model predicts an increased likelihood that AmNA-modified ASO would yield clinical efficacy, compared to IND-1233-ASO. However, such a conclusion would be speculative because observable high cerebral bioavailability is unlikely to be achieved in human cohorts, considering the method of administration used in animal studies.

Another approach was suggested by Hayashita-Kinoh et al. [47], who designed a specific alpha-synuclein ribozymes-carrying rAAV vector and showed that it was able to reduce alpha-synuclein protein levels in cultured cells and ameliorate neuronal cell loss in the SNpc of the MPP+ rodent model.

Specific microRNAs (miRNAs) represent an alternative molecular tool that can be employed for reducing the neuronal burden of alpha-synuclein in neurons of PD and DLB, although their therapeutic potential is not yet well assessed. However, according to a recent study, a brain-enriched miRNA miR-7 that downregulates alpha-synuclein production via interaction with the 3′ untranslated region of the encoding mRNA [48] ameliorates the pathology triggered by the inoculation of alpha-synuclein preformed fibrils (PFF) into mouse striatum. The adeno-associated virus 1-based vector (AAV-miR-7) was used for delivery, and the resulting expression of miR-7 in the striatum reduced the levels of both monomeric, including phosphorylated, and aggregated alpha-synuclein, attenuated neurodegeneration, neuroinflammation, and behavioral deficits that are typical for this mouse model of alpha-synucleinopathy [49].

### 2.2. Methods of Drug Delivery

Data obtained by several research groups have shown potential efficacy in reducing *SNCA* expression in PD; however, there are significant mechanistic and practical limitations that may prevent such therapies from reaching the clinical-trial stage of development. For instance, the method of delivery remains a significant practical limitation. With the presently developed technologies, for reliable and consistent reductions in *SNCA* expression, it is likely that delivery would have to be made directly into brain tissue and be repeated multiple times over a significant period. In addition, the direct delivery of siRNAs would involve targeting a specific disease-relevant neuroanatomical area, such as the SNpc. Therefore, this strategy would not prevent alpha-synuclein accumulation in non-treated areas. For instance, treating the SNpc may prevent the worsening of motor symptoms but would not prevent the progression of cognitive deficits caused by the involvement of higher cortical brain areas.

Parenchymal delivery of siRNAs has many practical limitations. However, it must be acknowledged that deep brain stimulation (DBS) is currently used in clinical practice to provide symptomatic relief of motor symptoms. DBS involves placing probes at or anatomically close to the SNpc [50]. This raises the possibility that the direct delivery of siRNA therapeutics could occur at the same time, thus providing both a symptomatic and a disease-modifying intervention. However, DBS is currently used in more advanced PD where siRNA technologies may not be appropriate and, at present, a solid probe is used [50]. Therefore, this proposal would be only worth considering if DBS occurred at the early stages of the disease and if a method of intraparenchymal delivery was developed (e.g., a probe with a cannula).

Specific peptides can also be used to improve siRNA delivery to the nervous system. Spencer et al. [51] developed an 11-amino acid peptide, based upon the apoB protein that successfully facilitated the transport of siRNA against alpha-synuclein across the BBB, thus achieving greater cerebral bioavailability. However, the translational potential of intravenous small peptides as a drug delivery mechanism remains to be seen, one of the main concerns being the presence of peptidases and proteases in serum/plasma, leading to degradation of the therapeutic peptide [52].

**Figure 2:**
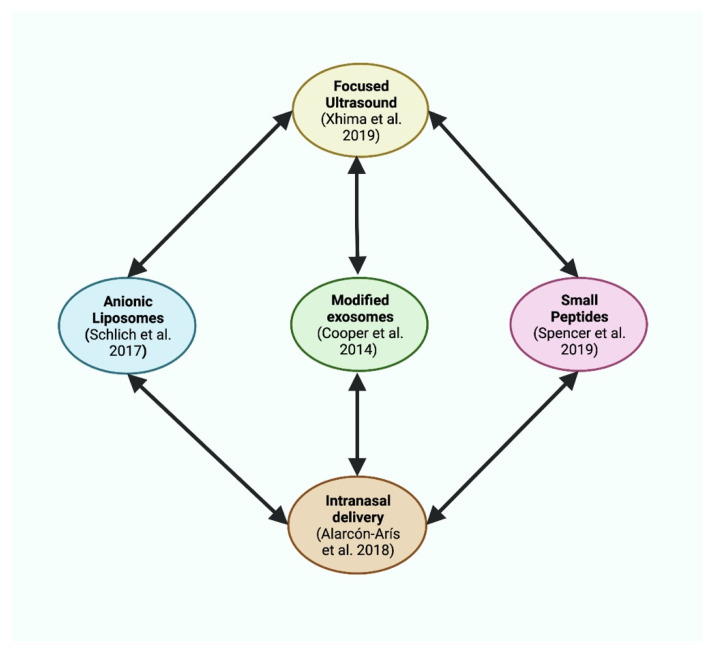
The varying novel strategies that could be utilized to improve RNAi BBB penetration. Each strategy is described in the main text, but it should be emphasized that the combination of some of these strategies (arrows) might be considered for further increasing treatment efficiency. This figure was created with BioRender.com.

It is now widely accepted that the development of novel drug delivery methods to facilitate blood-brain barrier (BBB) penetration is critical in developing an RNAi therapeutic that could be administered intravenously. Numerous potential strategies have been proposed to improve RNAi BBB penetration (Figure 2). Cooper et al. [53] showed that the intravenous infusion of a modified exosome, containing the central nervous system-specific rabies virus glycoprotein (RVG) peptide and siRNA targeting the *SNCA* mRNA, in the S129D alpha-synuclein rodent model could decrease the level of this mRNA in multiple brain areas, with no reported toxicity. The proposed ability of the RVG protein to improve blood-brain penetration has been attributed to its binding to nicotinic acetylcholine receptors on endothelial cells [54]. Anionic liposomes with RVG peptide and siRNA have also been shown to reduce alpha-synuclein in mouse primary hippocampal neurons [55]. Although liposomes might trigger a significant immune response, they are considered to be a more promising drug delivery vehicle because exosomes still present translational challenges, including isolation methods and the involvement of immune cells [56].

Another proposed strategy is the use of non-invasive magnetic resonance-guided focused ultrasound (mrgFUS), with an intravenous infusion of microbubbles containing an AAV-9 packaged shRNA against alpha-synuclein [57]. Using this strategy, the successful reduction of alpha-synuclein gene expression (by up to 60%) was achieved in the hippocampus, SNpc, olfactory bulb, and dorsal motor nucleus of a human alpha-synuclein-expressing rodent model. These results suggest that mrgFUS could provide a non-invasive methodology to enhance the efficiency of RNAi technologies. However, it must be acknowledged that the safety and efficacy of FUS have yet to be validated, with some authors suggesting that the FUS-mediated opening of the BBB could induce hemorrhagic, ischemic and inflammatory events [58].

The efficient reduction of alpha-synuclein expression in specific populations of mouse brain neurons by IND-1233-ASO [43] showed that intranasal delivery may also offer successful BBB penetration for RNAi-based drugs. Some argue that the internasal cavity provides improved cerebral bioavailability over systemic/oral drug delivery because of the ability to bypass hepatic first-pass metabolism, utilizing a semi-permeable endothelial membrane, high absorption area, and a faster absorption rate to target affected tissue [59]. However, it must be recognized that only small volumes of a particular drug can be administered via this route, and that the existence of intranasal peptidases and proteases still leads to a “pseudo” first-pass effect [60]. Currently, there is a lack of human evidence to establish whether or not internasal delivery offers superior cerebral bioavailability, compared to systemic delivery [61].

## 3. Immunotherapeutic Approaches for Alpha-Synuclein Degradation

The immunotherapeutic strategies devised to promote extracellular alpha-synuclein degradation and clearance are probably at the most advanced stage of development (Table 1). Here, these various immunotherapies will be defined as active or passive immunization strategies. The term “passive immunization” will refer to any antibody that directly targets extracellular alpha-synuclein, with a focus directed toward antibodies that target the C-terminal and the N-terminal domains of alpha-synuclein. In contrast, “active immunization” will refer to strategies designed to facilitate the patient’s own immune system in generating antibodies against pathological extracellular alpha-synuclein (e.g., vaccination).

### 3.1. Active Immunization

Considering that, at present, by the time a clinical diagnosis of PD is made there is already significant alpha-synuclein pathology, an active immunization approach is a particularly exciting prospect, as it may have the ability to protect at-risk groups or even the wider population from alpha-synuclein pathology before the onset of symptoms. Already, early attempts to induce the production of antibodies against alpha-synuclein in a transgenic mouse model via immunization with recombinant alpha-synuclein achieved the clearance of alpha-synuclein aggregates [67]. The most exciting development in active immunization regards the use of affitopes. These are small peptides that induce B-cell activation while being too small to elicit the potentially deleterious T-cell response [68]. The pharmaceutical company AFFiRiS has developed two Affitope vaccines (PD01A and PD03A), which, when administered to the PDGF-alpha-synuclein mouse model, showed reduced alpha-synuclein accumulation and improved motor deficits with no adverse effects or reduction in physiological alpha-synuclein [68]. In a Phase I trial of Affitope PD03A (NCT02267434), PD patients were given either high- or low-dose Affitope PD03A or a placebo [69]. Early results are promising, with no serious adverse side effects and an acceptable immune response against the vaccine and cross-reactivity against the alpha-synuclein-targeted epitope [70]. Recently, the results of a randomized, Phase I clinical trial for repeated PD01A vaccinations (four primary and two booster vaccinations) on patients with early PD (*n =* 32) have been released and showed an acceptable level of tolerability and safety. Furthermore, it was demonstrated that PD01A antibodies were observed in CSF, demonstrating successful target engagement [62]. However, it is worth noting that this Phase I trial does not have a double-blind component; however, this could be excused since no aspects of efficacy were being measured [62]. Regardless, AFFiRiS has announced plans to conduct a Phase II clinical trial in the near future [63]. However, if PD01A and PD03A do not show effectiveness in further clinical trials, other novel active immunization strategies have been developed. One example is a DNA-based vaccine that can target alpha-synuclein monomers prior to oligomerization [71]; however, this approach carries the risk of also reducing physiological alpha-synuclein, thus increasing the likelihood of side effects.

### 3.2. Passive Immunization

There is substantial preclinical evidence suggesting that a passive immunization strategy, with antibodies targeted toward the C-terminal domain of alpha-synuclein, may show clinical effectiveness. Indeed, the first study to show the feasibility of passive immunization against alpha-synuclein was with a monoclonal antibody that was directed toward the C-terminal domain of the protein. The administration of this antibody led to reduced alpha-synuclein accumulation and improved motor and cognitive deficits, in a PDGF-alpha-synuclein transgenic mouse model [72]. Further studies showed that monoclonal antibodies, including 9E4 (PRX002 murine homolog), could reduce alpha-synuclein accumulation and propagation, neurodegeneration, and motor deficits in alpha-synuclein transgenic mouse models [73,74]. It has also been shown that the humanized IgG1 monoclonal antibody PRX002 has a significantly greater affinity for pathological alpha-synuclein aggregates, compared to the monomer, with an acceptable BBB permeability [65]. Promisingly, this antibody has shown acceptable tolerability and a 95.5% reduction in serum alpha-synuclein in healthy volunteers in a Phase I clinical trial [64]. A multiple-ascending dose in a Phase 1b clinical trial of PRX002, consisting of three infusions on a monthly basis, also showed a promisingly high level of target engagement, once again with a 95.5% reduction in serum alpha-synuclein and BBB penetration, with dose-dependent rises of the PRX002 measurements of CSF in a sporadic PD cohort (*n =* 80) [65]. This is highly encouraging and a Phase II clinical trial (NCT03100149) to assess the efficacy, via the MDS-UPDRS score of monthly PRX002 infusions over a 52-week period in an early PD cohort (*n =* 316), is currently active [75].

MEDI1341 is another antibody that has been designed to target the C-terminal domain. Recently, Schofield et al. [76] demonstrated that MEDI1341 could prevent extracellular alpha-synuclein aggregation and cell-to-cell propagation in mice that underwent unilateral hippocampal alpha-synuclein injection. Furthermore, a Phase Ia clinical trial (NCT03272165), assessing the safety and tolerability of MEDI1341 in 48 healthy volunteers, has been completed, with the results expected to be published soon [77]. Additionally, a Phase Ib clinical trial (NCT04449484) involving 36 participants with PD is currently in the recruitment stage [78]. It is worth noting that Schofield et al. [76] also showed that an effector-null version of MEDI1341 not only had similar results to non-modified MEDI1341 but also reduced the likelihood of Fc-mediated effector cytotoxicity, due to the reduced risk of further activation of neuroinflammatory responses that might contribute to unwanted alpha-synuclein accumulation. Therefore, if MEDI1341 is shown to demonstrate unacceptable safety and efficacy levels, further study of an effector-null version of MEDI1341 may be warranted. However, the pharmaceutical company claims that MEDI1341 already demonstrates reduced effector function, leading to superior safety compared with the other novel antibodies being developed [79].

Antibodies directed toward the N-terminus of alpha-synuclein have also been developed. A human-derived antibody, BIIB054, showed a significant binding affinity preference toward aggregated alpha-synuclein, compared with monomeric alpha-synuclein (> 800 times). This molecule was shown to reduce alpha-synuclein accumulation in both a PFF-injected and AAV-A53T mouse model [80,81]. In the PFF-injected model, BIIB054 reduced the truncated alpha-synuclein spread by 52% to the contralateral cortex, while also improving motor deficits [81]. Promisingly, single-dose intravenous injections of this antibody at varying doses in a randomized Phase I clinical trial were shown to be safe and tolerable in a cohort that included both healthy and PD volunteers. In addition, the antibody could also be measured in the CSF (0.2% compared to plasma concentration) in the healthy participant cohort [66]. Consequently, a multicenter, randomized, double-blind, placebo-controlled Phase II clinical trial (NCT03318523) with an active-treatment dose-blinded period is currently active, with the aim of assessing the efficacy (measured by a change in MDS-UPDRS), safety, pharmacokinetics, and pharmacodynamics of BIIB054 [82].

An antibody specifically directed toward pathological oligomeric alpha-synuclein, which does not interact with physiological monomeric alpha-synuclein, would be an extremely useful compound. Lindstrom et al. [83] showed that the antibody mAb47 had a high specificity toward oligomeric alpha-synuclein protofibrils, with the ability to selectively lower their burden and reduce motor impairments in the Thy1-hA30P-alpha-synuclein transgenic mouse model. This consequently led to the development of ABBV-0805 (BAN0805), which was due to undergo a Phase I clinical trial (NCT04127695) but was later withdrawn for unspecified strategic reasons [84]. However, the search for an antibody with a high binding affinity to oligomeric alpha-synuclein continues. A very recent screening study identified four possible antibodies that have a stronger binding affinity to oligomeric compared with monomeric alpha-synuclein [85], and an earlier study showed that another five antibodies with specificity to oligomeric alpha-synuclein reduced the accumulation of these toxic species and lowered hippocampal neurodegeneration in mThy1-alpha-synuclein mice [86].

The most important information about translational studies and clinical trials, as described above, is collated in Supplementary Table S1.

## 4. Current Limitations, Challenges and Future Directions

### 4.1. Establishing the Therapeutic Window for Alpha-Synuclein Knockdown

**Figure 3:**
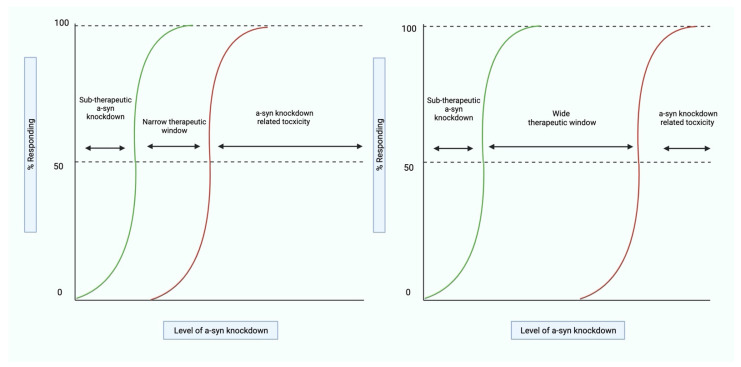
A therapeutic window of alpha-synuclein knockdown. The therapeutic window of alpha-synuclein knockdown needs to be defined to increase confidence in the safety and efficacy of these strategies. A sub-therapeutic level of alpha-synuclein knockdown would lead to further alpha-synuclein aggregation and related neurodegeneration. It is also possible that a toxic level of alpha-synuclein knockdown would lead to further SNpc neurodegeneration. The size of this therapeutic window has major implications for the future of alpha-synuclein knockdown strategies; with a narrow therapeutic window, it is increasingly difficult to achieve and retain therapeutic dosages of the proposed strategy. However, some have proposed that alpha-synuclein knockdown-related toxicity occurs beyond 90% [89], implying a wide therapeutic window. In clinical practice, to retain this therapeutic window, suitable alpha-synuclein knockdown therapeutic monitoring protocols may need to be developed. This would require access to validated biomarkers that ought to be sensitive, specific, and able to discriminate between physiological and pathological brain alpha-synuclein, which is a major challenge for the field. Additionally, to improve the clinical safety of alpha-synuclein knockdown, strategies to achieve a rapid reversal of alpha-synuclein knockdown-related toxicity would also need to be developed. This figure was created with BioRender.com.

The successful therapeutic reduction of pathological alpha-synuclein could also lead to unintentional reductions in normal physiological alpha-synuclein and potential alpha-synuclein knockdown-related side effects. Some data suggest that the complete knockdown of physiological alpha-synuclein can contribute to neurodegeneration in the SNpc and, therefore, could further worsen Parkinson’s-related neuropathology. The administration of shRNA has led to nigrostriatal degeneration and dopaminergic loss in both rodent and non-human primate models [87,88]. The precise level of alpha-synuclein knockdown before toxicity occurs has yet to be established; however, it is likely to be greater than 90% [89]. Furthermore, the systemic administration of these therapeutics would be the most practical. It must be considered that alpha-synuclein is highly expressed in some peripheral tissues, such as red blood cells (RBCs) [90]. However, the potential extra-cerebral effects of alpha-synuclein knockdown are still unknown and warrant further investigation. Therefore, it can be deemed critical that the tolerable but effective level of alpha-synuclein knockdown (the therapeutic window) is established before these strategies progress to the clinical trial stages of development (Figure 3).

However, the translational applicability of studies demonstrating alpha-synuclein knockdown-related toxicity still remains controversial. For instance, Zharikov et al. [91] showed that the chronic unilateral administration of AAV-delivered shRNA, targeted toward *SNCA* to the SNpc of WT rodents over a 12-month period, did not lead to substantial neurodegeneration. There was a small decrease in TH expression, but the authors claimed that this was not related to alpha-synuclein knockdown and could be attributed to the methodology employed (e.g., vector delivery and cellular transduction). Furthermore, many of the above-discussed strategies involving RNA interference technology have not shown neurodegeneration in either rodent or non-human primate models [39,40]; however, it could be argued that these studies only achieve therapeutic levels of alpha-synuclein reduction. It is important to note, here, that all the above studies involved the use of viral vectors that might have an unpredictable effect. However, a full germline knockout of alpha-synuclein also does not cause any obvious changes to general physiology, and mild changes to the nigrostriatal system were revealed only in aged, 2-year-old mice [24,92]. Although this finding might be explained by developmental compensation for the complete loss of alpha-synuclein in mice with a conventional knockout of the *SNCA* gene, tamoxifen-induced conditional inactivation of this gene in the neurons of 6-month-old mice also did not show significant changes one year later, i.e., in 18-month-old animals [93]. However, inactivation of the gene in the neurons of older, 12-month-old mice caused potentially harmful changes in dopamine turnover in the striatum [87]. This could suggest that older PD treatment groups are more susceptible to alpha-synuclein knockdown-related effects on the SNpc. The applicability of this data is unknown, but it should be noted that in the UK, the highest incidence rate of new Parkinson’s diagnoses is in the 75–79 age category, the second-highest being 80–84 [94]. Therefore, the success of alpha-synuclein post-transcriptional therapeutic strategies may be dependent on earlier identification and diagnosis of PD and the corresponding alpha-synuclein pathology. This may require a biomarker with a high predictive value for PD, in addition to a biomarker that can identify early alpha-synuclein pathology. Furthermore, a mass screening program would need to be initiated in order to successfully identify candidates who would be suitable for early therapeutic intervention.

### 4.2. Limitations Specific to Immunotherapies

Considering that Phase II clinical trials in both passive and active immunizations are either being planned or are currently underway, a discussion on the potential limitations of immunotherapies against alpha-synuclein as a DMT in PD is worthwhile. The first challenge to consider for active immunization is that numerous vaccinations—including booster vaccinations—are required, increasing both the cost- and time investment of these types of therapies. One further consideration is that extracellular alpha-synuclein disassembly might lead to the release of significant alpha-synuclein-soluble products, and whether this phenomenon might be followed by deleterious consequences is unknown. Furthermore, there is always the concern of an abhorrent immune response to active immunization; however, the recently designed Affitopes appear to mitigate this problem.

Next, the heterogeneity of alpha-synuclein forms as a result of various post-translational modifications could lead to certain strains being resistant to passive immunization with anti-alpha-synuclein antibodies. Furthermore, the potential intracellular pathological consequences due to the escape of certain strains from antibody-targeted degradation are unclear, as the relative contribution of various strains to neuropathology has still not been completely established [95,96]. In addition, the vast majority of studies discussed above only attempted to demonstrate a higher binding affinity to oligomeric alpha-synuclein compared to monomeric alpha-synuclein and do not investigate the strain-specificity of the researched compound. One potential solution to increase confidence that the proposed antibody can target a broad range of pathological alpha-synuclein strains is to encompass a variety of rodent alpha-synuclein aggregation mouse models in the preclinical testing of the compound. From a biomarker perspective, even though it is currently unfeasible, subcategorization of PD cases according to the relative proportions of alpha-synuclein strains, while also understanding the specificity of different immunotherapies to various alpha-synuclein strains, could allow clinicians to offer a more personalized and, therefore, efficacious immunotherapeutic intervention.

The true clinical relevance of extracellular alpha-synuclein is yet to be clarified. Extracellular alpha-synuclein is involved in the neuron-to-neuron propagation of alpha-synuclein across the brain; however, the majority of the pathological consequences of alpha-synuclein aggregation occur in the intracellular space. Therefore, the efficacy of these therapeutics is highly dependent on their ability to prevent the prion-like spread of alpha-synuclein to non-affected neurons and brain areas. However, it must be considered that extracellular alpha-synuclein does also contribute to the activation of microglia and to a subsequent deleterious neuroinflammatory response that does contribute to PD pathology [97]. Thus, simultaneously targeting pathological alpha-synuclein accumulation in both the extracellular and intracellular compartments may be required before any disease-modifying effect can be observed.

### 4.3. Optimizing Preclinical Data

At present, there are no rodent or non-human primate models that fully encapsulate both the pathology and symptomology of sporadic PD. It must be noted that, as yet, no DMT has been fully shown to be efficacious in a PD cohort; therefore, the predictive validity of these models cannot be determined. Despite this, testing on animal models of alpha-synuclein aggregation is currently the best strategy to aid the discovery and development of novel therapeutics. Animal alpha-synuclein aggregation models can be defined using three broad categories: transgenic rodent models, viral-vector delivery rodent models, and preformed fibril-injection models. A brief summary of the various alpha-synuclein aggregation models and their respective advantages and disadvantages is presented in Table 2.

The majority of studies discussed in this review are usually reliant on one alpha-synuclein aggregation animal model. A single alpha-synuclein aggregation model is unlikely to capture the heterogeneity of both pathological drivers and symptoms within a PD cohort. Therefore, the therapeutic value of treatments solely tested on a single alpha-synuclein aggregation model is likely to be overestimated. Preclinical research should utilize strategies to minimize such a risk, including testing the therapeutic on numerous different models. Furthermore, the endpoints used in preclinical research vary from group to group. Ideally, different groups would use similar endpoints in the same rodent models, allowing for a more valid preclinical comparison between the potential efficacies of different therapeutics. One such approach would be for all research groups to adopt the same validated, highly protocolized drug development pathway, for example, as suggested by Koprich et al. [98]. Progression along such a preclinical drug development pipeline should also be dictated by the same pre-agreed endpoints. A common drug development strategy would allow preclinical comparisons between therapeutics, thus allowing for a more informed selection of candidate drugs to progress toward human clinical trials.

### 4.4. Enhancing Clinical Trial Design

Clinical trials must at least achieve two principal definable concepts within drug development: proof of principle (PoP) and proof of concept (PoC). PoP is a concept whereby the proposed drug (administered at tolerable doses) can lead to a quantifiable improvement in disease-relevant biomarkers. PoC would equate to a therapeutic demonstrating a lasting disease-modifying effect within a PD cohort. Current clinical trials are reliant on changes in baseline between treatment and placebo groups in the components of the widely accepted MDS-unified Parkinson’s disease rating scale (MDS-UPDRS) scoring system. This will not show the short-term symptomatic effects of these disease-modifying treatments. It is critical to ascertain whether these interventions will actually worsen symptoms in the short term. This would allow clinicians to adequately inform patients and implement changes in pre-existing symptomatic medications, to negate the negative short-term effects of these therapeutics. Another perspective is that the use of the MDS-UPDRS scoring system could be too rigorous an endpoint to demonstrate PoC in a sporadic PD cohort; more broad primary endpoints should also be considered. In addition, it has not yet been defined what degree of difference in the MDS-UPDRS change from baseline is required before a disease-modifying effect can be confirmed.

Additionally, a carefully chosen, optimized cohort is more likely to show clinical efficacy for the proposed disease-modifying strategies. It must be acknowledged that there is a significant variety of clinical manifestations in PD, involving varying degrees of both motor and cognitive symptoms. Furthermore, within a sporadic PD cohort, there will be a range of intercellular contributors to PD neuropathology. One possible solution is to form a sporadic PD cohort, whose disease pathology is primarily driven by alpha-synuclein aggregation. This would require a sensitive and specific biomarker to brain alpha-synuclein aggregation (for comprehensive reviews of potential alpha-synuclein pathology-related biomarkers, see [99,100,101]). Due to the heterogeneity of the disease pathology, the current clinical trials of the proposed therapeutic strategies may include subgroups of individuals within a treatment group wherein the drug does indeed show a disease-modifying effect. The groups currently conducting clinical trials should thus identify the presence of these subgroups within their treatment cohort and aim to define common biological or clinical characteristics within that subgroup. By defining those subgroups that respond to treatment, researchers can narrow the exclusion/inclusion criteria for future clinical trials and, therefore, increase the likelihood that the therapeutic will prove efficacious in a clinical trial study.

It is a distinct possibility that some of the therapeutics discussed here will only show a disease-modifying effect if used in a prodromal PD cohort. However, to conduct a Phase II clinical trial in this type of cohort, the group would need a high likelihood of both developing PD and that the major pathogenic driver for their PD-related pathology will be alpha-synuclein accumulation. Again, there will be a role for biomarkers that are both predictive of PD development and specific to alpha-synuclein-related pathology. However, it is more likely that a multi-factorial approach will be required to identify such groups, including the consideration of many possible disease-relevant risk factors. Therefore, at present, this type of trial would be both timely and expensive, with a low likelihood of efficacy if poor cohort selection is conducted. However, a study of this type of cohort may be of interest if the Phase II clinical trials that are currently underway show efficacy in a symptomatic PD cohort.

## 5. Conclusions

In summary, there is a significant body of evidence suggesting that alpha-synuclein is a contributor to both the pathogenesis and progression of neuropathology in PD. Multiple anti-alpha-synuclein therapeutic strategies have been proposed. It is evident that strategies targeting post-transcriptional alpha-synuclein are currently at an early preclinical stage of development, with numerous translational barriers to overcome before in-human clinical trials can be seriously considered. Comparatively, strategies targeting alpha-synuclein extracellular degradation are at a far more advanced stage of clinical development, with Phase II clinical trials currently in progress. However, these diametrically opposed strategies have common and definable translational barriers. In the future, it is critical that these translational barriers are addressed to improve the feasibility of using anti-alpha-synuclein therapeutics in a real-world PD cohort (summarized in Supplementary Table S2). Regardless, therapeutically targeting alpha-synuclein aggregation is a valid approach to developing a disease-modifying treatment in sporadic PD. Certainly, the results of forthcoming Phase II clinical trials will be a critical point in this research field, potentially confirming both the efficacy and safety of this approach.

## Figures and Tables

**Table 1:** A summary of the status/results of previous and ongoing clinical trials involving the immunotherapeutic targeting of extracellular alpha-synuclein degradation.

Author/Clinicaltrial.gov Identifier	Compound	Study Type	Status/Results
Active Immunization			
NCT02267434	PD03A	Phase Ib clinical trial in an early PD cohort (*n =* 36)	No serious adverse side effects, acceptable immune response against the vaccine, cross-reactivity against alpha-synuclein-targeted epitope
Volc et al., 2020 [62]	PD01A	Phase Ib clinical trial in an early PD cohort (*n =* 32)	PD01A antibodies were observed in CSF, demonstrating successful target engagement, acceptable levels of tolerability and safety
Affiris. 2021 [63]	PD01A	Phase II clinical trial (cohort type not yet specified)	Intention expressed
**Passive Immunization**			
Schenk et al., 2017 [64]	PRX002	Phase Ia clinical trial (*n =* 40)	Acceptable safety and tolerability; 95.5% reduction in serum alpha-synuclein
Jankovic et al., 2018 [65]	PRX002	Phase Ib clinical trial in a mild to moderate PD cohort (*n =* 80)	Acceptable safety and tolerability; 95.5% reduction in serum alpha-synuclein and BBB penetration, dose-dependent rises of PRX002 measurements of CSF.
NCT03100149	PRX002	Phase II clinical trial in an early PD cohort (*n =* 316)	Active
NCT03272165	MEDI1341	Phase Ia clinical trial (*n =* 49)	Completed, awaiting results
NCT04449484	MEDI1341	Phase Ib clinical trial in a mild to moderate PD cohort (*n =* 36)	Recruiting
NCT04127695	ABBV-0805 (formerly BAN0805)	Phase Ia clinical trial (*n =* 0)	Withdrawn for unspecified strategic reasons
Brys et al., 2019 [66]	BIIB054	Phase Ib clinical trial, including a healthy (*n =* 48) and early PD cohort (*n =* 18)	Acceptable safety and tolerability; drug measured in the CSF (0.2% compared to plasma concentration)
NCT03318523	BIIB054	Phase II clinical trial in an early PD cohort (*n =* 357)	Active

**Table 2:** Advantages and disadvantages of alpha-synuclein aggregation animal models.

Models	Positives	Negatives
Transgenic rodent model (e.g., Thy1-hA30P-alpha-synuclein)	Construct validity: gain-off-function *SNCA* mutations occur in human PD cohorts Content validity: widespread brain alpha-synuclein accumulation occurs Can be used to model cognitive deficits	Face validity: substantial SN neurodegeneration does not occur; therefore, modeling motor deficits is challenging Expresses only one form of alpha-synuclein aggregate. Does not model the heterogeneity of various alpha-synuclein strains in the human PD Expresses significantly greater levels of WT and/or mutant alpha-synuclein than the human PD brain
Viral-vector delivery rodent model (e.g., AAV-hA53T)	High construct and face validity: area-specific alpha-synuclein accumulation, reduced dopamine release, striatal neurodegeneration, and motor impairments.	Expresses significantly greater levels of WT and/or mutant alpha-synuclein than the human PD brain The neuroanatomical specificity of the vector insertion means that widespread neurodegeneration is not possible; it is thus difficult to model cognitive impairments
PFF injection rodent model	Face validity: alpha-synuclein accumulation, neurodegeneration, and varying degrees of motor and cognitive deficits, depending upon the area of the initial injection High construct validity contains many strains of alpha-synuclein, and recombinant PFFs can be sourced from post-mortem PD brain samples. Propagation of alpha-synuclein accumulation beyond the injection site; models the alpha-synuclein spread to other cortical areas and the associated onset/worsening of motor and/or cognitive symptoms	Construct validity is dependent on the validity of the prion-like hypothesis and Braak hypothesis The appearance of motor deficits is variable.
WT non-human primate model	Used for assessing the pharmacokinetic/pharmacodynamic profile of the therapeutic Used for assessing the safety/tolerability of the therapeutic	No disease-relevant pathology, so we cannot assess the efficacy of the therapeutic
Viral-delivery non-human primate model (e.g., AAV1/2-hA53T)	Monitoring protocols involving novel biomarkers can be refined and optimized Can assess potential efficacy, unlike the WT NHP	A relatively new model, so the exact role in anti-alpha-synuclein drug development is currently unknown Time- and cost-intensive

## Data Availability

Not applicable.

## Supplementary Materials

The following are available online. Two supplementary tables are published online alongside the manuscript: Table S1: Summary of some of the most significant findings and limitations of the key papers/clinical trial announcements in the field of therapeutics against synthesis and the extracellular degradation of alpha-synuclein in Parkinson’s disease. Table S2. Future directions/research priorities.
