# Supplementary material for: Toward a Disease-Modifying Therapy of Alpha-Synucleinopathies: New Molecules and New Approaches Came into the Limelight
Source: Molecules. 2021 Dec 3;26(23):7351. doi: 10.3390/molecules26237351 (PMC8658846; doi:10.3390/molecules26237351)
Supplement: Supplementary file 1 [file molecules-26-07351-s001.zip › molecules-1493678-supplementary.pdf]

Table S1. Summary of some most significant findings and limitations of the key papers/clinical trial announcements in the field of therapeutics against synthesis and extracellular degradation of alpha-synuclein in Parkinson's disease.

Reference/ Clinicaltrial.gov identifier	Compound	Model used/Study type	Findings	Limitations
**Synthesis**				
Sapru et al. 2006 [35]	lentiviral-delivery of shRNA	Human dopaminergic cell line (SH-SY5Y), LV-alpha-synuclein-shRNA-CMV-EGFP vector injected into the striatum of WT rats.	Reduced *SNCA* mRNA/expression	No quantification of change in alpha-synuclein aggregation, no measure of changes in behavioural deficits
Lewis et al. 2008 [38]	siRNA, shRNA, naked siRNA	*Cornu Ammonis* (CA1) of the hippocampus of mice	Reduced *SNCA* mRNA/expression	No quantification of change in alpha-synuclein aggregation, no alpha-synuclein aggregation model used therefore no measure of changes in behavioural deficits
Zharikov et al. 2015 [36]	AAV-mediated delivery shRNA	WT rats, rotenone rat model	35% reduction in alpha-synuclein expression, reduced motor deficits, reduced striatal dopaminergic loss in rotenone rodent model.	No pharmacokinetic/pharmacodynamic analysis, decrease in TH expression in WT rats
Alarcón-Arís et al. 2018 [39]	ASO+indatraline IND-1233-ASO	WT mice	Lowered expression of endogenous alpha-synuclein mRNA in the brainstem areas/increased dopaminergic and serotonergic signalling without neurodegeneration, Intranasally delivered	No detailed pharmacokinetic/pharmacodynamic analysis, no alpha-synuclein aggregation model used therefore no measure of motor/behavioural deficits
Pavia-Collado et al. 2021 [41]	IND-1233-ASO	A30P/A53T-alpha-synuclein mice (tg)	Reduced alpha-synuclein synthesis in the SNpc and LC, improved DA signalling	No changes in performance on motor and behaviours batteries
Uehara et al. 2019 [42]	(AmNA)-modified ASO	TH-SNCA-140 m, Thy1-alpha-synuclein mice (tg)	Lowered alpha-synuclein mRNA and protein levels, improved motor deficits	No detailed pharmacokinetic/pharmacodynamic analysis, Intracerebral administration of compound.
Spencer et al. 2019 [45]	11aa based upon apoB protein	PDGF-alpha-synuclein mice (tg)	Reduced alpha-synuclein expression and aggregation, decreased neurodegeneration, systemically administered	Peptide-based compound have risk of serum peptidases/proteases degradation
McCormack et al. 2010 [37]	21-base pair siRNA duplex against the squirrel monkey *SNCA*	Squirrel monkey	Lowering of alpha-synuclein expression by 50% in dopaminergic neurons in SN	Administered via intracerebral method, no model of alpha-synuclein aggregation used.
Cooper et al. 2014 [47]	RVG+ siRNA exosome	S129-alpha-synuclein (tg) mice	Decreases alpha-synuclein mRNA	No measurement of motor/behavioural deficits
Schlich et al. 2017 [49]	RVG + siRNA lipsomes	Mouse primary hippocampal neurons	Decreased alpha-synuclein expression, serum stability	No disease relevant rodent model used therefore no motor/behavioural deficits
Xhima et al. 2018 [51]	Focused ultrasound+ microbubbles of AVV9 shRNA	Human alpha-synuclein expressing rodent model	60% of alpha-synuclein gene expression no change in TH markers	No detailed pharmacokinetic/pharmacodynamic analysis, no measure of motor/behavioural deficits, safety concerns surrounding FUS
**Active Immunisation**				
Masliah et al. 2005 [61]	Full-length recombinant α-syn expressed in E. Coli	PDGF-alpha-synuclein mice (tg)	Decreased alpha-synuclein aggregation, increased clearance of alpha-synuclein	Multiple alpha-synuclein aggregation overexpression models would be preferable, no detailed pharmacokinetic/pharmacodynamic analysis. No measure of motor and cognitive deficits
Mandler et al. 2014 [62]	PD01A and PD03A	PDGF-alpha-synuclein and mThy1-alpha-synuclein mice (tg)	Reduced alpha-synuclein accumulation, neurodegeneration, improved motor deficits with no adverse effects, reduction in physiological alpha-synuclein.	Study could further test PD01A and PD03A on non-human primates to increase confidence in drug safety
NCT02267434	PD03A	Phase Ib clinical trial in early PD cohort	No serious adverse side effects, acceptable immune response against	n/a

			the vaccine, cross reactivity against alpha-synuclein targeted epitope	
Volc et al. 2020 [56]	PD01A	Phase Ib clinical trial in early PD cohort (n=32)	PD01A antibodies were observed in CSF, demonstrating successful target engagement, acceptable tolerability and safety	n/a
**Passive Immunisation**				
Masliah et al. 2011 [66]	monoclonal antibody	PDGF-alpha-synuclein mice (tg)	Reduced alpha-synuclein accumulation and improve motor/cognitive deficits	Multiple alpha-synuclein aggregation overexpression models would be preferable
Bae et al. 2012 [67]	274-antibody	PDGF-alpha-synuclein mice (tg)	Microglial dependent alpha-synuclein clearance and reduced cell-to-cell propagation, improved motor deficits	Multiple alpha-synuclein aggregation overexpression models would be preferable
Games et al. 2014 [68]	9E4 (PRX002 murine homolog)	Thy1-alpha-synuclein mice (tg)	Reduced alpha-synuclein accumulation, reduce neurodegeneration via inhibiting alpha-synuclein truncation, reduced motor and cognitive deficits	Multiple alpha-synuclein aggregation overexpression models would be preferable
Schenk et al. 2017 [58]	PRX002	Phase Ia clinical trial	Acceptable safety and tolerability 95.5% reduction in serum alpha-synuclein	n/a
Jankovic et al. 2018 [59]	PRX002	Phase Ib clinical trial with PD cohort	Acceptable safety and tolerability 95.5% reduction in serum alpha-synuclein and BBB penetrance, dose-dependent rises of PRX002 measurements of CSF.	n/a
NCT03100149	PRX002	Phase II clinical trial with an early PD cohort	Active	n/a
Schofield et al. 2019 [70]	MEDI1341	Unilateral hippocampal alpha-synuclein injection into rats and cynomolgus monkeys	Lowered extracellular alpha-synuclein aggregation	No measure of motor/cognitive deficits
NCT03272165	MEDI1341	Phase Ia clinical trial	Completed, awaiting results	n/a
NCT04449484	MEDI1341	Phase Ib clinical trial 36 participants with PD	Recruiting	n/a
Lindstrom et al. 2014 [77]	mAb47	Thy1-A30P-alpha-synuclein mice (tg)	High specificity towards oligomeric alpha-synuclein protofibrils with the ability to selectively, lower alpha-synuclein protofibrils and reduced motor impairments, no toxic adverse effects	Study could further test mAb47 on non-human primates to increase confidence in drug safety
NCT04127695	ABBV-0805 (formerly BAN0805)	Phase Ia clinical trial	Withdrawn for unspecified strategic reasons	n/a
El-Agnaf et al. 2017 [80]	Syn-O1, -O2, and -O4, Syn-F1 and -F2	Thy1- alpha-synuclein mice (tg)	Reduced alpha-synuclein accumulation and lowered hippocampal neurodegeneration in	No measure of motor/cognitive deficits, Multiple alpha-synuclein aggregation overexpression models would be preferable
Tran et al. 2014 [96]	Syn303	PFF treated mice	Rescue alpha-synuclein propagation and reduce motor deficits, reducing dopaminergic neuronal cell loss	Motor deficit still occurred, further test Syn303on non-human primates to increase confidence in drug safety
Shahaduzzaman et al. 2015 [74]	N-terminus AB1	AAV-WT alpha-synuclein rodent model	Reduced alpha-synuclein in SN, Reduced dopaminergic neuronal cell loss, Reduced motor deficits	Multiple alpha-synuclein aggregation overexpression models would be preferable
Weihofen et al. 2019 [75]	BIIB054	PFF-injected and AAV-A53T mouse model	Reduce alpha-synuclein accumulation in both models, Reduced loss of DATs in the striatum. improved motor deficits	Study could further test BIIB054 on non-human primates to increase confidence in drug safety
Brys et al. 2019 [60]	BIIB054	Phase Ib clinical trial including both healthy and PD participants	Safe and tolerable, Drug measured in the CSF (0.2% compared to plasma concentration)	n/a
NCT03318523	BIIB054	Phase II clinical trial	Active	n/a

Table S2. Future directions/research priorities

- New methods of drug delivery to improve blood-brain barrier drug penetrance for post-transcriptional alpha-synuclein strategies.
- An antibody with a high binding affinity for oligomeric alpha-synuclein.
- Investigate the alpha-synuclein strain specificity of immunotherapeutics.
- Clarify if targeting extracellular alpha-synuclein alone will yield a disease-modifying effect in a real-world Parkinson's disease (PD) patient cohorts.
- Establish the therapeutic window for alpha-synuclein knockdown.
- Develop a specific and sensitive biomarker to alpha-synuclein brain accumulation for diagnostic, staging and drug monitoring purposes.
- Form a protocolised and standardized preclinical drug-development pathway for anti alpha-synuclein therapeutics.
- Define what difference in MDS-UPDRS change from baseline is required before a disease-modifying effect can be confirmed.
- Identify responder subgroups within a treatment group in the forthcoming Phase II clinical trials.
- Develop strategies to enhance the selection of clinical trial sporadic PD cohorts, potentially based on alpha-synuclein pathology related biomarkers.
- Consider the utility of anti alpha-synuclein therapeutics in a prodromal PD cohort.
